# Appearance comparisons and body image in women's everyday lives

**DOI:** 10.1186/2050-2974-3-S1-O20

**Published:** 2015-11-23

**Authors:** Jasmine Fardouly, Lenny Vartanian

**Affiliations:** 1 UNSW Australia, Australia

## 

Appearance comparisons are an important socio-cultural factor influencing body dissatisfaction among young women. These appearance comparisons can occur when interacting with another person, reading magazines, watching television, or when engaging with social media. However, little is known about the frequency and outcome of appearance comparisons through these different mediums in women's everyday lives. We conducted an Ecological Momentary Assessment study in which female undergraduate students (n = 150) completed a brief online survey at five random times every day for five days. In this survey, participants were asked if they had made an appearance comparison. If they had, they were also asked what medium they compared themselves through and how they rated compared to that person (i.e., more attractive, less attractive). All participants then completed state measures of mood, appearance dissatisfaction, and intention to diet and exercise. Participants reported comparing themselves most often to others in person, and in person comparisons were associated with less negative outcomes (e.g., less body dissatisfaction) than were comparisons through other mediums (e.g., social media). These findings suggest that appearance comparisons are not always bad, but it is specifically comparisons to idealised images (whether through traditional or social media) that is associated with negative outcomes.

